# Supplementary material for: Identification of photoperiod-induced specific miRNAs in the adrenal glands of Sunite sheep (Ovis aries)
Source: Front Vet Sci. 2022 Jul 22;9:888207. doi: 10.3389/fvets.2022.888207 (PMC9354845; doi:10.3389/fvets.2022.888207)
Supplement: Supplementary file 1 [file Data_Sheet_1.ZIP › Supplementary file/miRDeep2 file.zip!/miRDeep2 file/Know_Structure/SP42A1/oar-mir-432.pdf]

miRBase precursor : oar-mir-432
Total read count : 972
oar-miR-432 read count : 968
remaining reads : 4

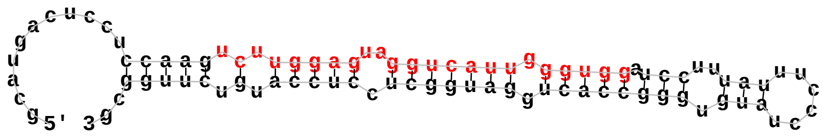

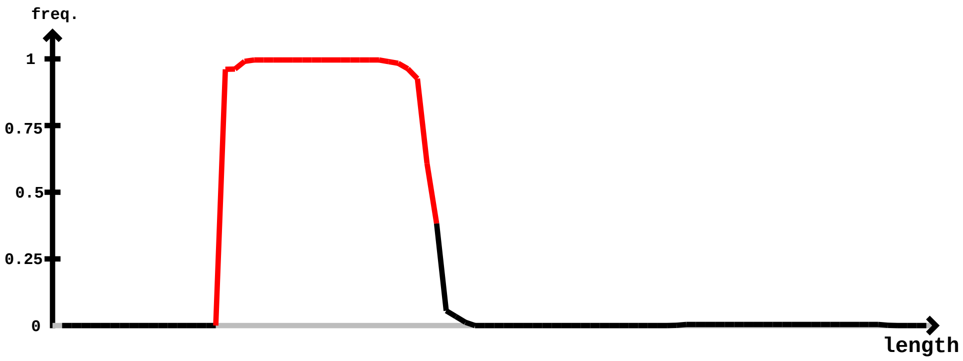


```
                                oar-miR-432
5'- gcaugacuccuccaagucuuggaguaggucauuggguggauccuuuauuucccuaugugggccacuggauggcuccuccaugucuuggcg   -3'    exp
    ..........(((((.(.(((((..(((((((.(((((.(((..(((......))).)))))))).))))))).))))).).)))))..    reads      mm         sample
    ................ucuuggaguaggucauA.........................................................    1          1          seq
    ................ucuuggaguaggucauu.........................................................    5          0          seq
    ................ucuuggaguaggucauug........................................................    6          0          seq
    ................ucuuggaguaggucauugg.......................................................    20         0          seq
    ................ucuuggaguaggucauuggg......................................................    36         0          seq
    ................ucuuggaguaggucauugggu.....................................................    308        0          seq
    ................ucuuggaguaggucauugggug....................................................    219        0          seq
    ................ucuuggaguaggucauuggguggg...................................................    313        0          seq
    ................ucuuggaguaggucauuggguggga..................................................    20         0          seq
    ................ucuuggaguaggucauugggugggau.................................................    5          0          seq
    ................ucuuggaguaggucauugggugggauc................................................    1          0          seq
    .................cuuggaguaggucauugggu.....................................................    1          0          seq
    ..................uuggaguaggucauuggguggg...................................................    5          0          seq
    ..................uuggaguaggucauuggguggga..................................................    1          0          seq
    ..................uuggaguaggucauugggugggau.................................................    13         0          seq
    ..................uuggaguaggucauugggugggauc................................................    9          0          seq
    ...................uggaguaggucauugggugggau.................................................    3          0          seq
    ...................uggaguaggucauugggugggauc................................................    2          0          seq
    ...............................................................acuggauggcuccuccaugucu.....    1          0          seq
    ................................................................cuggauggcuccuccaugucu.....    2          0          seq
    ................................................................cuggauggcuccuccaugucuu....    1          0          seq
```
